# A Review and Meta-Analysis on Altered Brain Structure in Patients Born with Non-Syndromic Cleft Lip and/or Palate

**DOI:** 10.1177/10556656251327526

**Published:** 2025-03-24

**Authors:** N. N. Homoud, A. J. Ireland, M. Sherriff, Z. AlSaffar, A. J. V. Davies, J. R. Sandy

**Affiliations:** Bristol Dental School, 1980 The University of Bristol, Bristol, UK

**Keywords:** meta-analysis, NSCL/P, brain, CL/P, CP, CLO, review

## Abstract

**Objective:**

To determine if there is evidence for a relationship between the presence of a non-syndromic cleft lip and/or palate (NSCL/P) and altered brain structure in cleft affected individuals.

**Design:**

Electronic database (MEDLINE; EMBASE; Cochrane library) and manual searches were performed and were limited to English language texts published between 1st of January 1969 until 1st of June 2024. Data extraction and risk of bias assessment were carried out independently by two reviewers. A meta-analysis on 9 publications was performed using a random effects model.

**Main outcome measure:**

Comparison of brain structure between patients born with cleft lip and/or palate and unaffected healthy individuals.

**Results:**

The review identified 11 studies, of which 9 were included in the meta-analysis. The latter comprised 398 individuals with NSCL/P compared to 458 unaffected controls. There was statistical evidence to suggest a reduction in the overall intracranial volume and the total cerebellar volume in the NSCL/P cases compared to the controls (Hedges’ *g* and 95% confidence intervals: −0.36 [95% CI: −0.65, −0.08] and −0.69 [95% CI: −0.84, −0.53], respectively). For the cortical gray matter, straight gyrus, and total cerebral volume, there was no statistical evidence to suggest a difference between the NSCL/P cases and the controls.

**Conclusion:**

In individuals with NSCL/P, the overall intracranial volume and the total cerebellar volume were both smaller than in unaffected controls.

## Introduction

It is estimated that in every 1000 births, between 1 and 2 children are born with cleft lip and/ or palate (CL/P).^1^ The etiology is multifactorial and commonly a result of the interaction between genetic and environmental factors.^2^ Phenotypic expression is a cleft lip, a cleft palate, or both.^1^ Cleft lip and palate are more common in males (2:1), while cleft palate only is more common in females (2:1).^3^ Up to 30% of children born with CL/P have their orofacial cleft as part of a well-defined syndrome, while the remaining 70% occur as an isolated anomaly.^4^

Children born with CL/P may have associated problems throughout their lives, including early struggles with feeding, speech, and hearing, as well as longer term issues with their facial appearance and bullying. Cognitive issues may be seen in syndromic CL/P, but are less pronounced in non-syndromic CL/P (NSCL/P). Nevertheless, there have been reports that subjects with NSCL/P have a lower than average IQ,^5–7^ have greater difficulties with expressive language,^8–10^ and reduced social functioning.^11,12^ Recent work on IQ has found that those born with NSCL/P are generally within the average Full Scale Intelligence Quotient (FSIQ) range but in the lower end of normative data. FSIQ has been found to be statistically lower than controls in some studies.^13^ A large population-based registry study in Sweden found surprisingly that educational outcomes for girls with cleft are more negatively affected than for boys with cleft. There was no difference in outcomes for boys with cleft when compared to the male population.^14^ Poor clinical outcomes for children born with cleft have a cumulative effect on educational attainment and interventions (either clinical or in school) are likely to be needed to remediate these difficulties.^15^ Despite earlier studies raising concerns on poor cognition in those born with cleft^16,17^; in non-syndromic clefting, there is no genetic reason for this. With supportive education and good clinical outcomes, educational attainment remains unaffected.^18,19^

The brain and face are both derived from the neuroectoderm and their development is linked in both normal and pathologic conditions.^20^ It is possible then that orofacial clefts might be associated with anomalies in brain structure, and some research has suggested this.^5,21–26^ There are possibilities that early and repeated exposure to anesthetics as well as reduced brain oxygenation (through obstructive sleep apnea) might also have an impact on neuronal development.^27^ Generally, altered brain structure is found to correlate with reduced cognitive performance.^23^ Studies on brain imaging are expensive to conduct and there are few available but summarizing the data and findings as well as understanding the implications is important. The aim of this review and meta-analysis was to explore the available evidence and determine if there is a relationship between the presence of NSCL/P and altered brain structure.

## Methods

### Search Strategy

A systematic search of the literature was conducted in the following electronic databases: MEDLINE, EMBASE, and the Cochrane library. The search was limited to English language only and the publications included were from January 1969 until June 2024. The following keywords were used in the search: “Cleft lip/” OR “Cleft palate/” AND “Phenotype/” OR “IQ” OR “BRAIN”. The reference lists of the included articles were also searched manually. Authors were contacted when clarification or additional data were required.

### Eligibility Criteria

In order to develop the research question, the Population, Exposure, Comparator, Outcome (PECO) framework was implemented. The participants included patients born with cleft lip and palate, live births, any ethnic group, both sexes, without any other associated syndromes, and participants could be involved in hospital or community settings. Exposure assessed the brain structure in both groups with healthy comparators. The outcome measured the presence of difference in brain structure between the cleft lip and palate and healthy individuals and if there is alteration in the quality of life.

### Study Selection

Within the PECO framework,^28^ the following criteria for the review were implemented: any study assessing brain structure in individuals born with CL/P, all ethnicities, either sex, and the cleft should not be associated with any syndrome. Additionally, any publication in the English language was included. Any study that included individuals with associated syndromes or were stillborn were excluded.

Two reviewers (NH and ZA) screened all the study titles and abstracts identified from the search that met the inclusion criteria. Any disagreements as to inclusion were resolved by a third reviewer (AI).

### Protocol

This review was performed according to the Cochrane Handbook^29^ and reporting was according to the Preferred Reporting Items for Systematic Reviews and Meta-Analysis (PRISMA) statement.^30^ The risk of bias was assessed using the Critical Appraisal Risks Programme (CASP) tool.^31^

### Outcome Measures and Statistical Analysis

A random effects meta-analysis was conducted to estimate the effect of CL/P on the brain structure from the selected studies using Stata 17 MP.^32^ Mean differences and their upper and lower 95% confidence intervals (CI) were chosen as effect sizes (Hedges’ *g*) and described with Forest plots. The data were analyzed by subgrouping different structures of the brain, namely: total cerebellar volume; cortical gray matter; total cerebral volume; intracranial volume; and straight gyrus. The heterogeneity between the studies was estimated using *τ*^2^ along with *I*^2^. To visualize potential bias, a contoured funnel plot was used. These plots have contours of statistical significance to aid interpretation. Where studies are missing in areas of little statistical evidence, any asymmetry maybe due to publication bias, whereas when missing in areas of strong statistical evidence then publication bias is less likely the cause of the asymmetry.^33^

## Results

**Figure 1.**
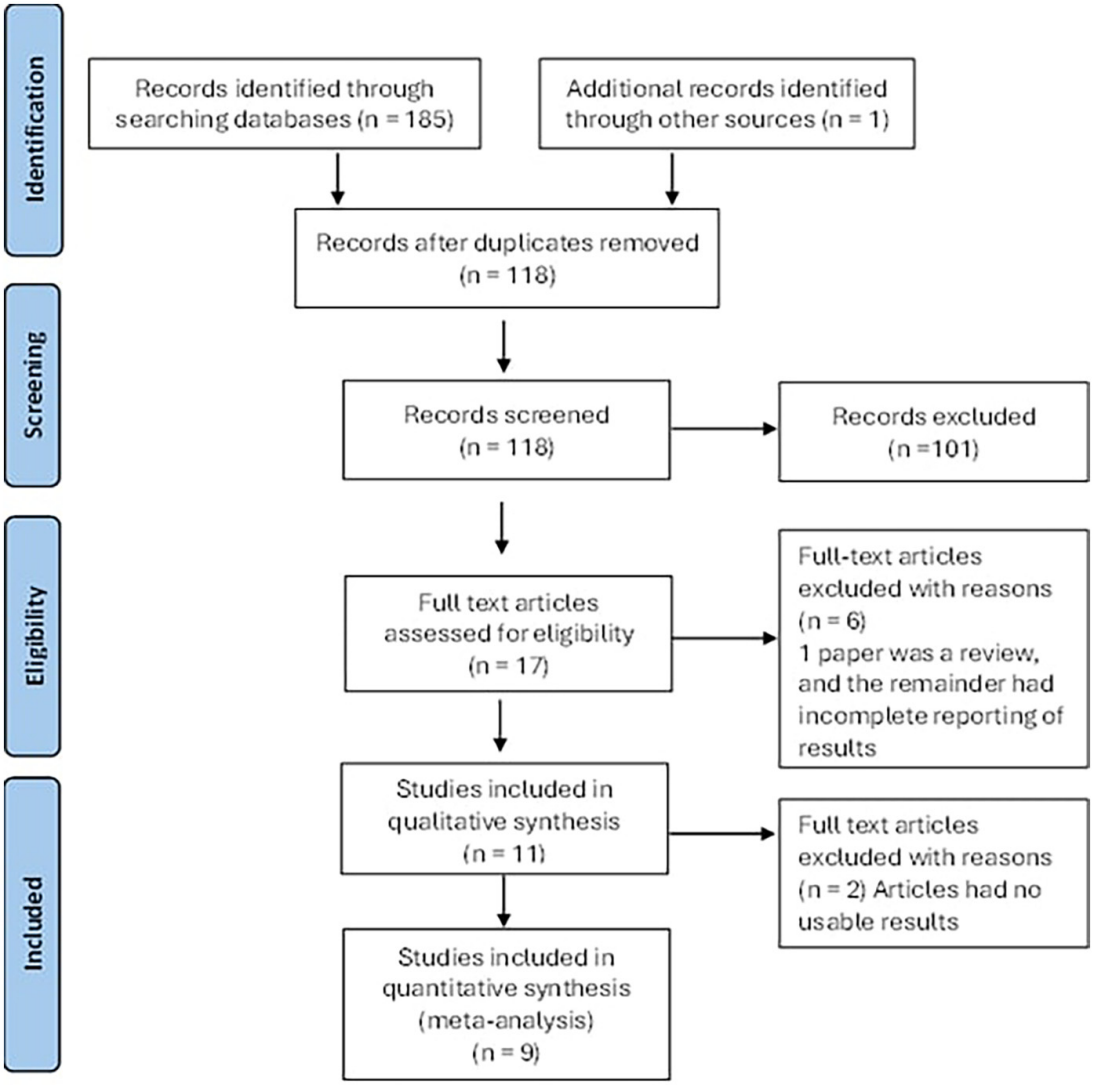
PRISMA (Preferred Reporting Items for Systematic Reviews and Meta-Analysis) flow diagram showing the article selection and reasons to eliminate papers.

The PRISMA flowchart (Figure 1) illustrates the article selection and elimination process. Initially 186 articles were identified through the search, and following deduplication there were 118. Screening of the titles and abstracts lead to the exclusion of a further 101 articles. Of the 17 remaining, a further one was excluded as it was a review. The final 16 articles underwent full data extraction. Five of these were subsequently excluded as the results were not clearly outlined. Using the remaining 11 papers, 5 different subgroups of assessment of brain structure were created for consideration, namely:

- Cortical gray matter (cm^3^);
- Straight gyrus (cm^3^);
- Intracranial volume (cm^3^);
- Total cerebellar volume (cm^3^);
- Total cerebral volume (cm^3^).

Of the 11 articles (Table 1), only 9 were used for the meta-analysis due to missing data (mean and standard deviation).

**Table 1.** Summary Characteristics of Papers Included in the Qualitative and Quantitative Analyses.

Study ID	Author, date, study design	Cleft group	Control group	Type of cleft and number of individuals	Outcome measures
**1**	Nopoulos et al.^22^ Case–control	74	74	CL (*n* = 18), CL/P (*n* = 33), and CP (*n* = 23)	Intracranial volume; total cerebellar volume; total cerebral volume
**2**	Van der Plas et al.^34^ Case–control	33	57	Right UCL ± P (*n* = 14) and left UCL ± P (*n* = 19)	Excluded from meta-analysis
**3**	Nopoulos et al.^33^ Case–control	46	46	BCL ± P (*n* = 11), right UCL ± P (*n* = 3), left UCL ± P (*n* = 18), and CP (*n* = 14)	Cortical gray matter; intracranial volume; total cerebellar volume; total cerebral volume
**4**	Devolder et al.^35^ Case–control	107	127	CL (*n* = 22), CL/P (*n* = 54), and CP (*n* = 31)	Total cerebellar volume
**5**	Adamson et al.^36^ Case–control	26	26	No data reported	Excluded from meta-analysis
**6**	Nopoulos et al.^37^ Case–control	14	14	CL (*n* = 1), CL/P (*n* = 8), and CP (*n* = 5)	Intracranial volume; total cerebellar volume; total cerebral volume
**7**	Conrad et al.^5^ Case–control	43	43	CL (*n* = 6), CL/P (*n* = 25), and CP (*n* = 11)	Total cerebellar volume
**8**	Conrad et al.^38^ Case–control	26	57	CL (*n* = 6), CL/P (*n* = 10), and CP (*n* = 10)	Total cerebellar volume; total cerebral volume
**9**	Boes et al.^24^ Case–control	30	43	CL (*n* = 8), CL/P (*n* = 15), and CP (*n* = 7)	Straight gyrus
**10**	Nopoulos et al.^21^ Case–control	46	46	BCL ± P (*n* = 11), right UCL ± P (*n* = 3), left UCL ± P (*n* = 18), and CP (*n* = 14)	Straight gyrus
**11**	Bodoni et al.^39^ Case–control	12	12	Right CLP (*n* = 1), left CLP (*n* = 4), and BCLP (*n* = 7)	Cortical gray matter; intracranial volume

Abbreviations: CL, cleft lip only; CL/P, cleft lip and palate; CP, cleft palate; BCL ± P, bilateral cleft lip with or without cleft of the palate; UCL ± P, unilateral cleft lip with or without cleft of the palate.

Out of the 11 articles, 9 were undertaken at the University of Iowa, USA, and they were case–control studies and the principal examination was Magnetic Resonance Imaging (MRI) to assess the brain structure. Some of the papers also included cognitive assessments; however, these were not included within the meta-analysis.

Using the Critical Appraisal Risks Programme (CASP) tool, the papers included within this review were assessed as being at a moderate to high risk of bias. Possible reasons for this included potential selection bias through the use of local newspaper advertisements, which might only be accessible to certain members of the community, and limited information as to the examiner(s) selecting the participants for enrollment.

**Figure 2.**
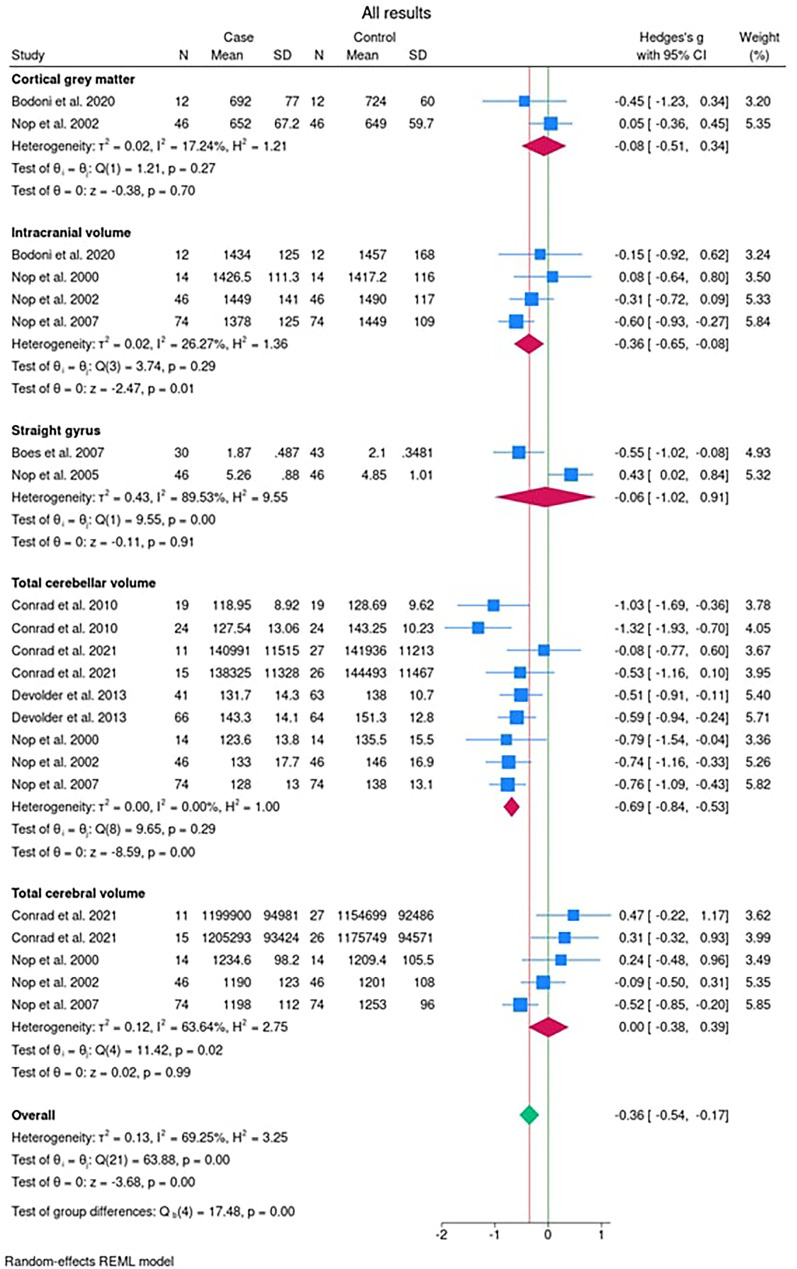
Forest plot of the data for each of the outcomes in the 9 studies listed sub-grouped in Table 1 (cortical gray matter, straight gyrus, intracranial volume, total cerebellar volume, and total cerebral volume).

After dividing the brain structure into the five subgroups (cortical gray matter; straight gyrus; intracranial volume; total cerebellar volume; and total cerebral volume), the results are presented as a composite forest plot (Figure 2). On the left of the plot is the study name. The line of no effect is highlighted in green. The red line indicates the overall effect of all areas of the brain which passes through the summary diamond for the overall results. Therefore overall, there is an effect of the presence of a cleft on brain structure (−0.36 [95% CI: −0.54, −0.17]). When considering each of the areas of the brain in turn:

### Cortical Gray Matter

There was no statistical evidence of an effect of the presence of NSCL/P on the structure of the cortical gray matter. However, one study described how cortical gray matter was increased in NSCL/P affected individuals.^33^

### Straight Gyrus

There was no statistical evidence of an effect of the presence of NSCL/P on the structure of the straight gyrus as shown by the summary diamond crossing the line of no effect (−0.06 [95% CI −1.02, 0.91]).

### Intracranial Volume

The summary diamond is to the left of the line of no effect (−0.36 [95% CI −0.65, −0.08]), indicating there is moderate statistical evidence of a reduced intracranial volume among children and adults with a cleft in comparison with their non-affected peers. However, it is also worth noting that some caution is needed when interpreting this result as *I*^2 ^= 26.27% would suggest moderate heterogeneity of the data.

### Total Cerebellar Volume

Here, the summary diamond is once again to the left of the line of no effect (−0.69 [95% CI −0.84, −0.53]), suggesting there is strong statistical evidence of a reduced cerebellar volume amongst children and adults with a cleft in comparison with their non-affected peers.

### Total Cerebral Volume

The Forest plot in this case suggests there is no statistical evidence to suggest a difference in the total cerebral volume between NSCL/P affected individuals and the unaffected controls, with the summary diamond crossing the line of no effect (0.00 [95% CI: −0.38, 0.39]).

**Figure 3.**
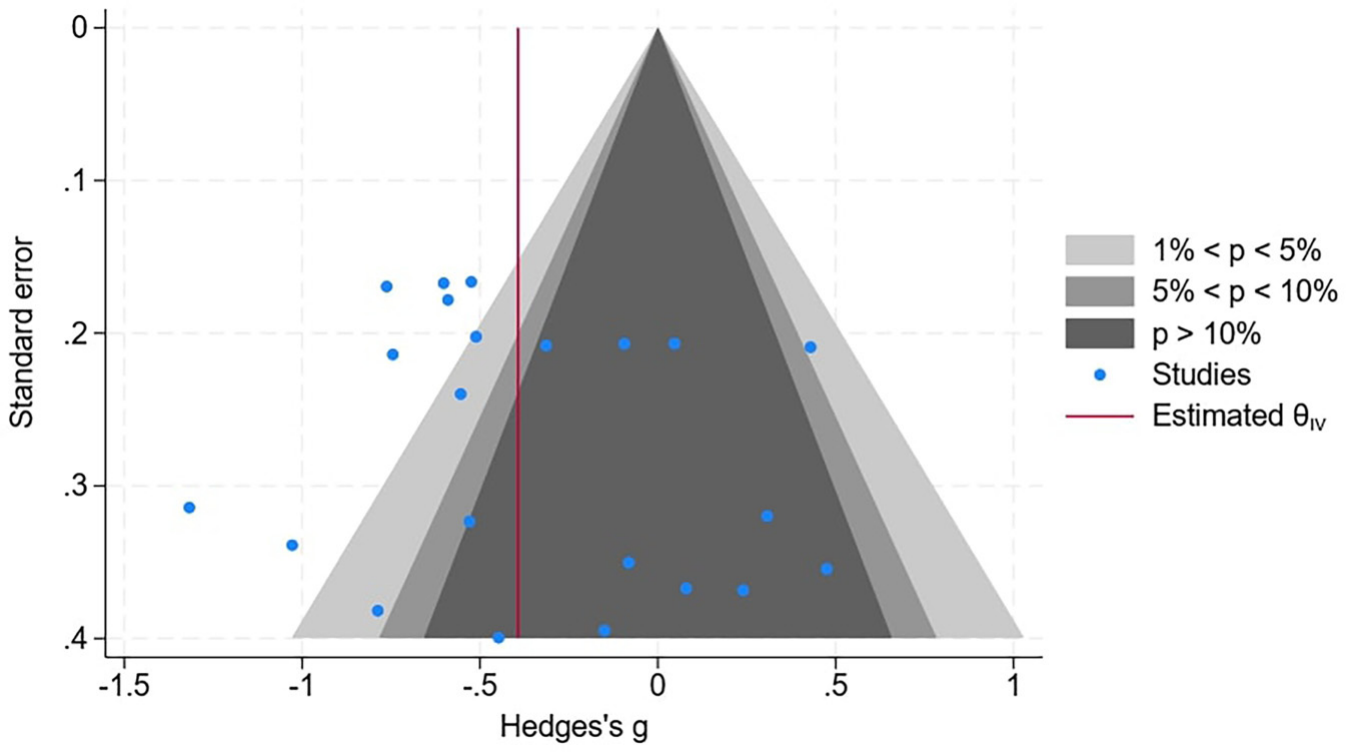
Contour-enhanced funnel plot for the assessment of publication bias. All 5 subgroups of brain structure are included within the 9 papers within the meta-analysis.

To help assess the risk of bias, a contoured funnel plot was used (Figure 3). This is essentially a scatterplot of the individual data points with respect to brain structure for each of the 9 papers used in the meta-analysis. It can be seen this scatter is asymmetric suggesting a degree of bias. The contour enhanced plot allows for statistical significance of study estimates to be considered and it would seem from the plot in Figure 3 that there are studies “missing” in the areas where nonsignificant studies would normally be plotted, suggesting publication bias based on statistical significance.

## Discussion

This review identified 11 studies, of which 9 were suitable for inclusion in the meta-analysis. The analysis considered 5 subgroups of brain structure for the ease of reporting which were: cortical gray matter, straight gyrus, intracranial volume, total cerebellar volume, and total cerebral volume. For 3 of these subgroups, cortical gray matter, straight gyrus, and total cerebral volume, there would appear to be no statistical evidence of an effect of clefting on the size of each structure when compared to unaffected controls. By contrast, there was statistical evidence to suggest an effect of the presence of cleft in the case of intracranial volume and total cerebellar volume. It is important to note that as this meta-analysis was conducted on retrospective, previously published data, there is a possibility of bias. Therefore, the findings might have over or underestimated the effect of the presence of cleft on altered brain structure. To help minimize selection bias, a strict protocol was adhered to following the creation a specific research question and related inclusion criteria utilizing the PECO approach. Inclusion was determined by two reviewers with a third used to help resolve any conflicts as suggested by Boutron et al.^40^ Potential bias was also formally assessed using a contour enhanced funnel plot, which showed a degree of asymmetry. This could have been not only due to publication bias based on statistical evidence,^41^ but also selective outcome reporting, selective or inadequate analysis, or poor methodological design.^42,43^ Certainly, when comparing the results, the demographics of the cleft groups were sometimes different (eg, all male participants in one, mixed sexes in another). Another factor that was encountered was age, with some studies including subjects over 18 years of age, whereas others only included children, even though all of the studies included in the meta-analysis were from the University of Iowa. This latter point may also suggest that the results might not be generalizable. Participants in the studies were excluded if they had an IQ below 70,^22,35^ which may have excluded NSCL/P affected individuals that had marked structural brain abnormalities perhaps related to their cleft. There were some small differences in the methodologies used in the papers: Nopoulos et al.^22^ evaluated the brain structure in NSCL/P patients and considered both males and females; Nopoulos et al.^37^ and Nopoulos et al.^33^ evaluated the brain morphology in adult NSCL/P males; Devolder et al.^35^ looked at dissimilar CL/P phenotypes and the potential effect on the cerebellar structure in both males and females; Conrad et al. (2010)^5^ investigated whether or not speech is affected by altered structural differences in the cerebellar structure in NSCL/P male and female patients; and Nopoulos et al.^21^ and Boes et al.^24^ considered social function in boys with NSCL/P and the possible relationship with ventral frontal cortex morphology.

The growth and development of the human brain is lengthy, and even though 95% of the volume of the cerebrum has formed by the age of 5 years, changes still take place within the white and gray matter until puberty and early adulthood.^44^ Children will grow at different rates and so it is not unreasonable to expect to see some structural differences in the brains of developing children, particularly when compared to adults, irrespective of the presence of a cleft. In most of the reports, age was considered to be a covariate in order to minimize the effect of age on the brain size and morphology between different age groups.^45^ Sex might also be expected to have an effect on brain structure in those born with a cleft,^22^ particularly as there are known sex differences in conditions that specifically affect the brain such as autism, learning disabilities, attention deficit disorder, and dyslexia,^46^ all of which are more common in males. We already know that the incidence of CL/P is greater in males (2:1 relative to females) and that CP is more common in females. The studies included in this review do suggest some differences in brain structure with age and sex, and it is possible that this might have an impact on cognition. However, it is not obvious that these structural differences result in any cognitive and/or behavioral abnormalities.

There was a difference in reporting of cleft subtype across the studies included in the meta-analysis; however, each study reported the collective NSCL/P cases to the controls. Current literature suggests distinct differences in the etiology of cleft subtypes,^47^ which could potentially have an impact on the brain structure. Furthermore, the greatest volume was seen in the controls, followed by the cleft lip only group, cleft palate only (CPO) group and the smallest was seen in the cleft lip and palate (CLP) group.^22^ These results are similar to those reported by Weinberg et al.,^26^ where variations were found between CPO and CLP affected groups when compared to controls. Further analysis looking at brain structure across cleft subtypes would be of some benefit.

Another limitation of studying the effect of cleft on brain structure is that relatively few centers will have access to the facilities and funding to be able to investigate them using non-invasive MRI. The studies included in the review were all cross-sectional, case–control studies. A unique longitudinal study^48^ evaluated brain development in males and females born with NSCL/P and compared this to a control group. Using an accelerated longitudinal design, (where cross-sectional and longitudinal data allow a wide age range to be investigated over a short study period) participants from the Nopoulos 2007 study and additional new participants returned for repeat cognitive assessment and MRI.^48^ Those with NSCL/P showed lower volumes of cerebellar gray matter and subcortical matter, and also had IQ scores within a normative range albeit at the lower end. Interestingly males born with a cleft palate only showed clinically significant measures of language disorder. In line with previous studies,^49,50^ sex and cleft phenotype are an influence in brain structure and development. Moreover, biological factors rather than psychosocial influences appear to account for these differences. These findings can inform the development of effective educational support interventions primarily during the formative years.

Nevertheless, longitudinal observation and larger sample size investigations are necessary, and further analysis across racial and sex factors on brain development in patients with craniofacial clefts are needed. Importantly there are some early studies which used imaging and vocalization to evaluate brain structure and function in infants with isolated oral clefts before exposure to anesthesia.^27^ These unique studies will only be possible in well-funded research environments, but they will increasingly yield information of clinical relevance.

## Conclusions

This review and meta-analysis summarizes the findings of previous studies investigating the potential relationship between the brain structure and the presence of NSCL/P. The principal findings when compared to unaffected controls were:

- There was a significant effect of cleft on intracranial volume and total cerebellar volume.
- There was no statistically significant effect of cleft on cortical gray matter, straight gyrus, and total cerebral volume.

It has highlighted that NSCL/P affected individuals demonstrate areas of brain dysmorphia when compared to unaffected controls. The pattern of dysmorphology may vary by:

- Cleft phenotype;
- Sex;
- Age.
